# Differential effects of slow rewarming after cerebral hypothermia on white matter recovery after global cerebral ischemia in near-term fetal sheep

**DOI:** 10.1038/s41598-019-46505-0

**Published:** 2019-07-12

**Authors:** V. Draghi, G. Wassink, K. Q. Zhou, L. Bennet, A. J. Gunn, J. O. Davidson

**Affiliations:** Department of Physiology, The University of Auckland, Auckland, New Zealand

**Keywords:** Experimental models of disease, Hypoxic-ischaemic encephalopathy

## Abstract

It is widely believed that rewarming slowly after therapeutic hypothermia for hypoxic-ischemic (HI) encephalopathy can improve outcomes, but its impact on white matter injury after HI is unclear. Fetal sheep (0.85 gestation) received 30 min ischemia-normothermia (n = 8), or hypothermia from 3–48 h with rapid spontaneous rewarming over 1 h (ischemia-48 h hypothermia, n = 8), or 48 h with slow rewarming over 24 h (ischemia-slow rewarming, n = 7) or 72 h with rapid rewarming (ischemia-72 h hypothermia, n = 8). Ischemia was associated with loss of total and mature oligodendrocytes and reduced area fraction of myelin basic protein (MBP) and 2′,3′-cyclic nucleotide 3′-phosphodiesterase (CNPase; immature/mature oligodendrocytes) and increased microglia and astrocytes. Total numbers of oligodendrocytes were increased by all hypothermia protocols but only ischemia-72 h hypothermia attenuated loss of mature oligodendrocytes. All hypothermia protocols similarly increased the area fraction of MBP, whereas there was only an intermediate effect on the area fraction of CNPase. Microglia were suppressed by all hypothermia protocols, with the greatest reduction after ischemia-72 h hypothermia, and an intermediate effect after ischemia-slow rewarming. By contrast, induction of astrocytes was significantly reduced only after ischemia-slow rewarming. In conclusion, slow rewarming after hypothermia did not improve oligodendrocyte survival or myelination or suppression of microgliosis compared to fast rewarming, but modestly reduced astrocytosis.

## Introduction

Therapeutic hypothermia initiated in the first 6 hours after birth and continued for 72 h significantly reduces death or moderate to severe disability after hypoxic-ischemic encephalopathy (HIE) in term and near-term infants^1^. Critically, hypothermia was associated with improved long-term neurocognitive and motor impairment in survivors^2^. Although most of the parameters required for optimal neuroprotection with hypothermia including the window of opportunity, depth and duration are now well understood^3^, there is no human controlled evidence for the optimal rate of rewarming. There is accumulating evidence from preclinical models that much of the effects of rewarming are mediated by the total duration of hypothermia^4^. It is important to appreciate that although the focus of most studies of neuroprotection has been on neuronal loss, both clinically and experimentally, HIE at term is associated with significant white matter injury. The effect of the rate of rewarming on white matter injury is unknown.

The predominant clinical patterns of white matter injury include damage to the posterior limb of the internal capsule and injury of cortical white matter in a watershed distribution^5,6^. For example, in infants with moderate to severe HIE, abnormal signal intensity in the posterior limb of the internal capsule on magnetic resonance imaging (MRI) is highly associated with impaired independent walking at 2 years of age^6^. Similarly, in a cohort of 84 term infants with HIE with normal appearance of the basal ganglia and thalami on MRI, the severity of white matter injury was significantly correlated with cognitive, visual, language, behavioral and seizure problems^7^.

Consistent with this, 30 minutes of global cerebral ischemia in near-term fetal sheep was associated with profound loss of oligodendrocytes, loss and disruption of myelination and increased numbers of microglia in the intragyral and periventricular white matter^4,8,9^. Therapeutic hypothermia started 90 minutes after global cerebral ischemia significantly improved white matter recovery, but the benefits were markedly attenuated when the initiation of hypothermia was delayed to 3 or 5.5 hours^9,10^. We have recently shown that compared with hypothermia starting 3 hours after ischemia and continued until 72 hours, 48 hours of hypothermia was associated with a marked deterioration of electrographic (EEG) power following rewarming and reduced neuronal survival^4^. For these protocols, fetal sheep rewarmed spontaneously over approximately 1 hour. We then tested the possibility that slower rewarming might alleviate EEG deterioration after the 48 hour protocol^11^. Intriguingly, after hypothermia for 48 hours, controlled slow rewarming over 24 h at ~0.2 °C/h was associated with improved recovery of EEG power compared to rapid spontaneous rewarming, but inferior grey matter survival compared to rapid spontaneous rewarming at ~5 °C/h. Notably, both fast and slow rewarming after 48 h of hypothermia showed reduced histological neuroprotection compared to 72 hours of hypothermia with rapid rewarming, suggesting that the effect of slow rewarming was simply mediated by the longer total duration of cooling. In the present study, in the same experimental cohort^11^, we tested the hypothesis that the better electrographic recovery despite no change in neuronal survival with slow rewarming after 48 hours of hypothermia was mediated by improved white matter protection.

## Methods

### Fetal Surgery

We have previously published the pH, blood gas, temperature changes, electrophysiological data and neuronal survival from the same cohort of animals as the present study^11^. All procedures and methods were approved by the Animal Ethics Committee of The University of Auckland under the New Zealand Animal Welfare Act, and the Code of Ethical Conduct for animals in research established by the Ministry for Primary Industries, Government of New Zealand. The experiment has been reported in compliance with the ARRIVE guidelines^12^.

At 125–126 days gestation (term is 145 days), 40 ewes underwent surgery for fetal instrumentation under sterile conditions. Food, but not water, was withdrawn 18 hours before surgery. Holding rooms were maintained at a constant temperature of 16 ± 1 degrees Celsius (°C) and 50 ± 10 percent (%) humidity on a 12 hour (h) light/dark cycle (light hours 06:00–18:00). Ewes were administered long acting oxytetracycline at 1 milliliter (mL)/10 kilogram (kg) (20 mg/kg, Phoenix Pharm, Auckland, New Zealand) intramuscularly (i.m.) for prophylaxis. Anesthesia was achieved by intravenous (i.v.) propofol (5 mg/kg; AstraZeneca Limited, Auckland, New Zealand) and maintained with 2–3% isoflurane in oxygen (O_2_) after intubation (Bomac Animal Health, NSW, Australia). Ewes were ventilated and under constant isotonic saline infusion (at approximately 250 mL/h) during surgery. Trained anesthetic staff monitored maternal heart rate, blood pressure and depth of anesthesia. Following sterilization of primary surgical fields, a laparotomy was conducted along the linea alba, followed by a 5–6 centimeter (cm) uterine incision in an area free of cotyledons and in parallel to major uterine blood vessels to expose the fetus.

Polyvinyl catheters were placed in both fetal brachial arteries to measure mean arterial blood pressure (MAP). A catheter to the amniotic cavity was secured to the fetal shoulder. Subcutaneous electrocardiogram (ECG) electrodes (Cooner Wire Co., Chatsworth, California, USA) were sutured onto the right shoulder and the left fifth intercostal space for monitoring of fetal heart rate (FHR). To prevent vertebral blood supply to the carotid arteries during ischemia, the vertebral-occipital anastomoses were ligated^13,14^. Double-ballooned inflatable occluder cuffs made in-house of silicone tubing (Degania Silicone, Kibbutz Degania Bet, Israel) were placed bilaterally around the carotid arteries for induction of cerebral ischemia. Furthermore, the right carotid artery received an ultrasound flow probe (size 3 S, Transonic systems, Ithaca, NY) for measuring of carotid blood flow (CaBF).

Two pairs of electroencephalograph (EEG) electrodes made of 7 stranded stainless steel wire (AS633–7SSF; Cooner Wire Co.) were placed on either hemisphere over the parasagittal parietal cortex (10 and 20 mm anterior to bregma and 10 mm lateral). Electrodes were secured on the dura with cyanoacrylate glue and a reference earth wire sewn over the fetal occiput. Cortical impedance was measured by placement of a third pair of electrodes 5 mm laterally to the EEG electrodes (3SS F, Cooner Wire Co.). An additional pair of electrodes (Cooner Wire) was sewn into the nuchal muscle to record fetal electromyogram (EMG) activity. Extradural temperature was measured by inserting a thermistor (Incu-Temp-1; Mallinckrodt Medical, St. Louis, MO) over the parasagittal dura (30 mm anterior to the bregma). A second thermistor was placed into the esophagus for measuring of body temperature. A cooling cap made of silicon tubing (3 × 6 mm, Degania Silicone) was placed onto the fetal head and secured with sutures.

The fetus was returned to the uterus and warm, sterile saline (approximately 500 mL at 37 °C) was added to counteract loss of amniotic fluid. All leads were exteriorized through an incision made into the maternal flank. Gentamicin (80 mg Gentamicin, Pharmacia and Upjohn, Rydalmere, New South Wales, Australia) was administered to the amniotic cavity. Bupivacaine (10 ml of 0.5%) and adrenaline (AstraZeneca Ltd., Auckland, New Zealand) were administered onto the skin of the maternal laparotomy incision. A catheter was inserted into the maternal long saphenous vein for post-operative care and euthanasia.

### Post-operative care

Animals were housed together in individual metabolic cages with *ad libitum* access to food and water. Prophylactic antibiotics were administered i.v. to the ewe daily for 4 days after surgery (600 mg benzylpencillin sodium, Novartis Ltd, Auckland, New Zealand, and 80 mg gentamicin). Fetal wellbeing was monitored by continuous tracing of MAP, FHR, CaBF, and EEG activity as well as fetal blood sampling. Heparinized saline was continually infused (20 U/mL at 0.2 mL/h) to ensure that fetal catheters were maintained patent, whilst the maternal catheter was flushed daily with heparinized saline.

### Data recording

Data recording began 24 hours before the start of the experiment and continued for the remainder of the experiment. Data were recorded and saved continuously to disk for off-line analysis using custom data acquisition programs (LabView for Windows, National Instruments, Austin, Texas, USA). Arterial blood samples were taken for preductal pH, blood gas, base excess (Ciba-Corning Diagnostics 845 Blood gas analyzer and co-oximeter, Massachusetts, USA), glucose and lactate measurements (YSI model 2300, Yellow Springs, Ohio, USA). All fetuses had normal baseline biochemical values for their gestational ages.

### Experimental Protocol

Experiments began at 129 ± 1 day (d) of gestation. Experiments were generally conducted at 9:00 am. Blood samples were taken two, four, and six hours after the end of ischemia, and daily thereafter. Animals were randomized into five groups: sham ischemia followed by normothermia (sham-control, n = 9); ischemia followed by normothermia (ischemia-normothermia, n = 8); ischemia followed by 72 hours of hypothermia (ischemia-72 h hypothermia, n = 8); ischemia followed by 48 hours of hypothermia (ischemia-48 h hypothermia, n = 8); or ischemia followed by 48 hours of hypothermia and 24 hours of slow rewarming (ischemia-slow rewarming, n = 7).

Cerebral ischemia was achieved by inflation of both balloons of each silicone carotid occluder with varying volumes of sterile saline and double-clamping of balloon inflation lines with clamps. Occlusion was confirmed successful where a rapid fall in EEG activity and in CaBF, as well as a delayed increase in cortical impedance was observed. Occlusion was maintained for 30 minutes and terminated immediately thereafter by release of clamps. The return of at least 80% CaBF was used to confirm successful reversal of occlusion. Successful occlusions also showed continuing suppression of EEG background after occlusion (≤10 dB).

Cooling was initiated 3 hours after the end of occlusion by linking the cooling coil over the fetal scalp to a pump (Grant Tx150, Grant Instruments Ltd, Cambridge, England) in a cooled water bath and circulating cold water through the cooling coil. The target extradural temperature was 31–33 °C. Cooling was stopped at 72 hours in the ischemia-72 h hypothermia group, and 48 hours in both the ischemia-48 h hypothermia and slow rewarming groups. In the sham-ischemia and ischemia-normothermia groups, the water was not circulated and the cooling coil remained in equilibrium with fetal temperature.

In the ischemia-72 h hypothermia and ischemia-48 h hypothermia groups, rapid rewarming was induced by switching off the cooling machine and allowing temperature to spontaneously return to baseline. In the ischemia-slow rewarming group, fetuses were rewarmed over 24 hours with a computer-controlled linear increase in bath temperature up to 39 degrees, at 0.2 °C per hour.

Ewes and fetuses were euthanized with an overdose of sodium pentobarbitone (300 mg/mL, Pentobarb 300, Provet, New Zealand Pty Ltd) administered into the saphenous vein 7 days after ischemia. Numbers of fetuses per pregnancy, sex, fetal body weight, and individual organ weights (brain, lungs, heart, liver, spleen, kidneys and adrenal glands) were recorded. The brain was removed and fixed for histological assessment of cerebral injury.

### Histological analysis

*In situ* fixation of fetal brains was achieved by perfusion of carotid arteries with 500 mL of 0.9% saline solution followed by 1000 mL of 10% phosphate buffered formalin and embedded using a standard paraffin tissue preparation^13^. Coronal slices of 10 micrometers (μm) in thickness were cut and mounted on chromalum-gelatine coated slides. Slices were obtained from the level where the dorsal hippocampus becomes apparent.

### Immunohistochemistry

The tissue was deparaffinized in xylene (2 × 15 min). Tissue was rehydrated at decreasing concentrations of ethanol (100, 95 and 70%; 5 min each). Sections were washed 3 times for 5 minutes each in phosphate buffered saline (PBS; 3 × 5 min) (2 L of 10x PBS (0.1 M): 160 g NaCl, 4 g KCl, 28.8 g Na2HPO4, 4.8 g KH2PO4, pH 7.4). Antigen retrieval was carried out via the pressure cooker (2100 Antigen Retriever, Aptum, Southampton, England) method using a citrate buffer (dH_2_0 450 ml, citric acid 8 mL and sodium citrate 42 mL). Sections were washed in PBS (3 × 5 min each). Endogenous peroxidase activity was blocked using 3% hydrogen peroxide in methanol for 30 minutes at room temperature covered by tin foil to prevent exposure to light. Sections were washed in PBS (3 × 5 min each) and blocked with 300 microliters (μL) of 3% normal goat serum (NGS, Life Technologies Ltd, Auckland, New Zealand) in PBS.

Sections were incubated overnight at 4 °C in primary antibodies (1:200). These included mouse anti-ionized calcium-binding adapter molecule 1 (Iba-1, Cat# ab15690 Abcam, Cambridge, England) as a marker of microglia/macrophages; mouse anti-myelin basic protein (MBP, Cat# MAB381, Lot# NG1726107, Millipore, Burlington, Massachusetts, USA) as a marker of myelination, mouse anti-2′,3′-Cyclic-nucleotide 3′-phosphodiesterase (CNPase, Cat# ab6319, Abcam) as a marker of immature/mature oligodendrocytes; mouse anti-APC antibody (CC-1, Cat# ab16794, Abcam) as marker of mature oligodendrocytes; mouse anti-oligodendrocyte lineage transcription factor 2 (Olig-2, Cat# ab236540, Abcam) as a marker of total oligodendrocytes and rabbit anti-glial-fibrillary-acidic protein (GFAP, Cat# ab68428, Abcam) as a marker of astrocytes in 3% NGS.

After washing with PBS (3 × 5 min each), sections were incubated at room temperature for 3 hours in 1:200 biotinylated goat anti-mouse (Cat# BA-9200, Vector Laboratories, Burlingame, CA, USA, Iba-1, CNPase, MBP, CC-1, Olig-2) or anti-rabbit (GFAP, Cat# BA-1000, Vector Laboratories) secondary antibody in 3% NGS. Incubation in 1:200 ExtrAvidin horseradish peroxidase (Cat# E2886, Sigma-Aldrich Ltd, Auckland, New Zealand) was conducted for 2 hours at room temperature. 3,3 diaminobenzidine (Sigma-Aldrich, Ltd.) was used to visualize positive staining. The tissue was visualized by microscopy to limit background staining. Staining was stopped by immersion in PBS solution. Slides were then dehydrated in increasing alcohol concentrations (70, 95 and 100%; 5 min each) and xylene (2 × 15 min) before being mounted (Scharlab S.L., Barcelona, Spain) with coverslips.

### Imaging and analysis

Two sections per animal were imaged using the Nikon eclipse 80i light microscope (Nikon Instruments Inc., Tokyo, Japan) for each antibody. Iba-1, Olig-2 and CC-1 stained sections were imaged at 20x magnification, whilst MBP, GFAP and CNPase at 40x magnification using the NIS elements Basic Research software (Coherent Scientific, Hilton, Australia). Images were taken from the intragyral white matter tract of the first parasagittal gyrus (IGWM1) and second parasagittal gyrus (IGWM2) and the periventricular white matter (PVWM) from both hemispheres. A total of 4 fields of view were obtained for each region from each animal.

Immunohistochemistry images were quantified using Image J software (National Institute of Health, Bethesda, United States). Iba-1 + ve microglia/macrophages, GFAP + ve astrocytes, Olig-2 + ve oligodendrocytes and CC-1 + ve mature oligodendrocytes were quantified by manual counting of each positively labelled cell. Both resting (ramified) and active (amoeboid) microglia were included. Quantification of the area fraction present with staining of MBP, CNPase and GFAP was performed using the Auto (MBP) and Huang (CNPase) and Mean (GFAP) auto-threshold settings on Image J (National Institute of Health).

### Statistics

The effects of cerebral ischemia and hypothermia on physiological and histological parameters were assessed with a mixed-design ANOVA, using time or region as a repeated measure (SPSS v23, SPSS Inc., Chicago, Illinois, USA). When statistical significance was found, post-hoc comparisons were performed with univariate ANOVA and Tukey correction. Non-parametric data were assessed using the Kruskal–Wallis test. Statistical significance was reported when P < 0.05, and data are presented as mean ± standard error of the mean (SEM).

## Results

### Fetal demographic data, pH and blood gas analysis and temperature

There were no differences in sex or fetal body or organ weights or proportion of singletons between groups at post-mortem (Table 1). Ischemia-normothermia was associated with a significant reduction in brain weight compared to sham control (P < 0.05), which was significantly attenuated in the ischemia-72 h hypothermia group. There was no significant difference in brain weight in the ischemia-slow rewarming group and ischemia-48 h hypothermia group compared to ischemia-normothermia.

**Table 1:** Fetal parameters at post-mortem.

Groups	N	Fetus (g)	Brain (g)	Singletons: Twins	Female: Male
Sham-control	9	4930 ± 275	48.9 ± 1.9	8: 1	4: 5
Ischemia-normothermia	8	4575 ± 311	40.1 ± 1.5*	7: 1	5: 3
Ischemia-48h hypothermia	8	4263 ± 204	41.9 ± 1.7*	8: 0	6: 2
Ischemia-slow rewarming	7	4744 ± 247	43.0 ± 1.4*	7: 0	3: 4
Ischemia-72h hypothermia	8	4720 ± 145	44.9 ± 0.9#	8: 0	4: 4

Fetal parameters for the sham-control, ischemia-normothermia, ischemia-48h hypothermia, ischemia-72h hypothermia, and ischemia-slow rewarming groups. Weights are in gram (g). Data are mean ± SEM; between-group comparisons performed by ANOVA. *P < 0.05 vs. sham-control; ^#^P < 0.05 vs. ischemia-normothermia.

Hypothermia was associated with a significant decrease in brain temperature to 31.3 ± 0.2 °C in the ischemia-72 h hypothermia group and 32.3 ± 0.3 °C in the ischemia-48 h hypothermia and ischemia-slow rewarming groups, compared with 39.5 ± 0.1 °C in the ischemia-normothermia group (p < 0.05)^11^. Esophageal temperature decreased to 37 ± 1.0 °C during hypothermia (P < 0.05). In the ischemia-48h hypothermia and ischemia-72h hypothermia groups, the end of cooling was marked by a rapid increase in brain temperature at 48 and 72 hours, respectively. In contrast, in the ischemia-slow rewarming group, a gradual increase in brain temperature (0.2 °C/hour) was observed from 48 to 72 hours after ischemia.

### Immunohistochemistry

**Figure 1:**
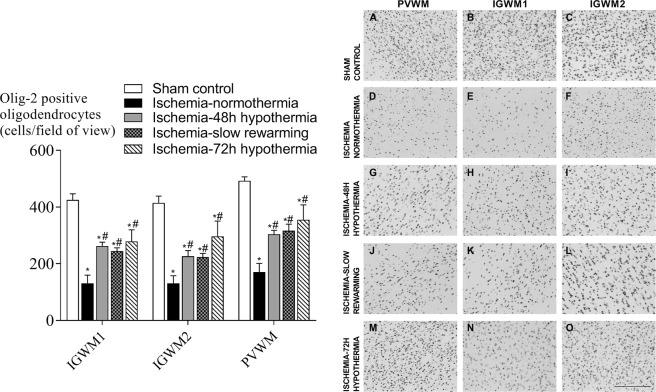
Total, Olig-2-positive, oligodendrocyte number (left) and photomicrographs (right) in the intragyral white matter of the first (**B**,**E**,**H**,**K**,**N**) and second (**C**,**F**,**I**,**L**,**O**) parasagittal gyri and periventricular (**A**,**D**,**G**,**J**,**M**) white matter in the sham control (**A–C**, n = 9), ischemia-normothermia (**D–F**, n = 8), ischemia-48 h hypothermia (**G–I**, n = 8), ischemia-slow rewarming (**J**–**L**, n = 7) and ischemia-72 h hypothermia (**M–O**, n = 8) groups. Data are mean ± SEM. *P < 0.05 vs sham control. ^#^P < 0.05 vs ischemia-normothermia. Scale bar 200 µm.

**Figure 2:**
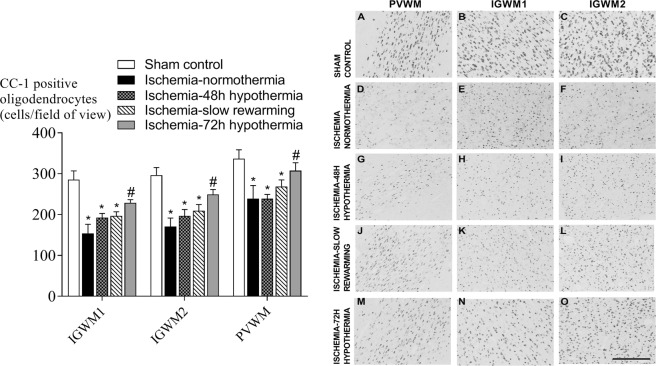
Mature, CC-1-positive, oligodendrocyte number (left) and photomicrographs (right) in the intragyral white matter of the first (**B**,**E**,**H**,**K**,**N**) and second (**C**,**F**,**I**,**L**,**O**) parasagittal gyri and periventricular (**A**,**D**,**G**,**J**,**M**) white matter in the sham control (**A**–**C**, n = 9), ischemia-normothermia (**D**–**F**, n = 8), ischemia-48 h hypothermia (**G–I**, n = 8), ischemia-slow rewarming (**J**–**L**, n = 7) and ischemia-72 h hypothermia (**M**–**O**, n = 8) groups. Data are mean ± SEM. *P < 0.05 vs sham control. ^#^P < 0.05 vs ischemia-normothermia. Scale bar 200 µm.

Ischemia-normothermia was associated with an overall reduction in the number of Olig-2 positive oligodendrocytes in the intragyral white matter of the 1^st^ (IGWM1) and 2^nd^ (IGWM2) parasagittal gyri and periventricular white matter (PVWM) compared to sham control (P < 0.05), which was partially attenuated by all hypothermia protocols (P < 0.05, Fig. 1). Ischemia-normothermia was also associated with a significant reduction in numbers of CC-1-positive mature oligodendrocytes compared to sham controls (P < 0.05, Fig. 2). Ischemia-72 h hypothermia was associated with a significant increase in numbers of CC-1-positive mature oligodendrocytes compared to ischemia-normothermia and was not significantly different to sham control, whereas ischemia-48 h hypothermia and ischemia-slow rewarming had no significant effect compared to ischemia-normothermia.

**Figure 3:**
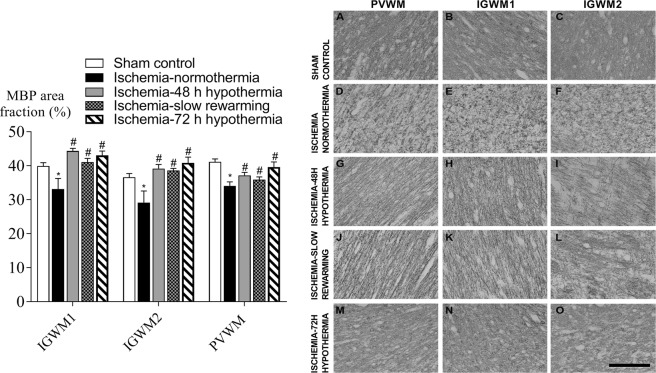
Area fraction of myelin basic protein (MBP, left) and photomicrographs (right) in the intragyral white matter of the first (**B**,**E**,**H**,**K**,**N**) and second (**C**,**F**,**I**,**L**,**O**) parasagittal gyri and periventricular (**A**,**D**,**G**,**J**,**M**) white matter in the sham control (**A–C**, n = 9), ischemia-normothermia (**D–F**, n = 8), ischemia-48 h hypothermia (**G–I**, n = 8), ischemia-slow rewarming (J-L, n = 7) and ischemia-72 h hypothermia (**M–O**, n = 8) groups. Data are mean ± SEM. *P < 0.05 vs sham control. ^#^P < 0.05 vs ischemia-normothermia. Scale bar 200 µm.

**Figure 4:**
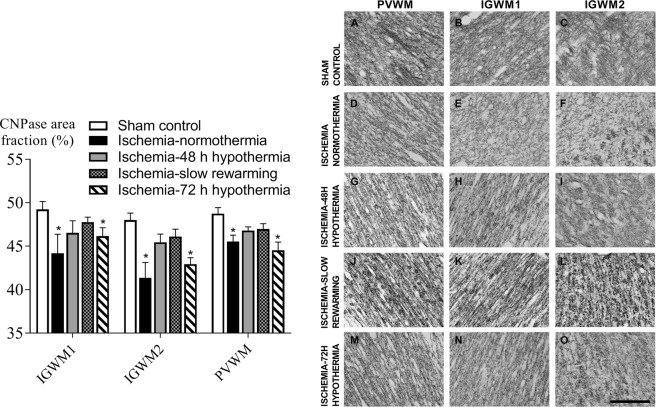
Area fraction of 2′,3′-cyclic nucleotide 3′-phosphodiesterase (CNPase, left) and photomicrographs (right) in the intragyral white matter of the first (**B**,**E**,**H**,**K**,**N**) and second (**C**,**F**,**I**,**L**,**O**) parasagittal gyri and periventricular (**A**,**D**,**G**,**J**,**M**) white matter in the sham control (**A–C**, n = 9), ischemia-normothermia (**D–F**, n = 8), ischemia-48 h hypothermia (**G–I**, n = 8), ischemia-slow rewarming (**J–L**, n = 7) and ischemia-72 h hypothermia (**M–O**, n = 8) groups. Data are mean ± SEM. *P < 0.05 vs sham control. Scale bar 200 µm.

Ischemia was associated with an overall reduction in the area fraction of MBP staining compared to control in all white matter regions (P < 0.05, Fig. 3). An increase in the area fraction of MBP labelling was seen in all hypothermia groups compared to ischemia-normothermia, to values that were not significantly different to sham controls. Similarly, ischemia was associated with a significant overall reduction in the area fraction of CNPase labelling (P < 0.05, Fig. 4). Hypothermia was associated with an intermediate area fraction of CNPase labelling, overall. Posthoc testing showed that the ischemia-48 h hypothermia and ischemia-slow rewarming groups were not significantly different to sham control or ischemia-normothermia, whereas the area fraction of CNPase labelling was significantly lower in the ischemia-72 h hypothermia group compared to sham control (P < 0.05).

**Figure 5:**
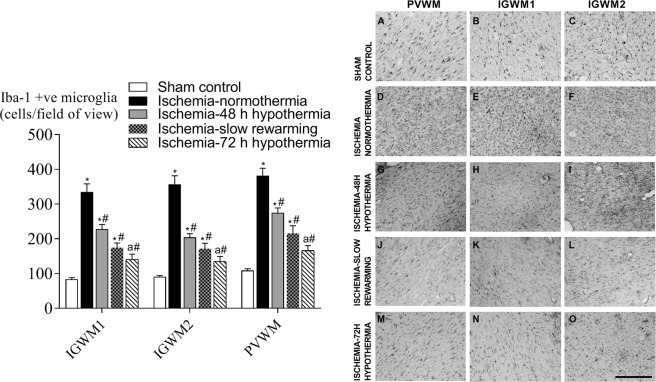
Ionized calcium-binding adapter molecule 1 (Iba-1) positive microglial number (left) and photomicrograph (right) in the periventricular white matter (**A**,**D**,**G**,**J**,**M**) and the intragyral white matter of the first  (**B**,**E**,**H**,**K**,**N**) and second parasagittal gyri (**C**,**F**,**I**,**L**,**O**) in the sham control (**A**–**C**, n = 9), ischemia-normothermia (**D**–**F**, n = 8), ischemia-48 h hypothermia (**G**–**I**, n = 8), ischemia-slow rewarming (J-L, n = 7) and ischemia-72 h hypothermia (**M**–**O**, n = 8) groups. Data are mean ± SEM. *P < 0.05 vs sham control. #P < 0.05 vs ischemia-normothermia. a P < 0.05 vs ischemia-48 h hypothermia. Scale bar 200 µm.

Ischemia was associated with an overall increase in the number of Iba-1 positive microglia in all white matter regions compared to sham control (Fig. 5, p < 0.05). Microglial number was significantly attenuated by all hypothermia protocols compared to ischemia-normothermia but remained significantly higher than sham control in the ischemia-48 h hypothermia and ischemia-slow rewarming groups. Ischemia-slow rewarming showed an intermediate reduction in microglial number, that was not significantly different to ischemia-48 h hypothermia or ischemia-72 h hypothermia. Ischemia-72 h hypothermia was associated with the greatest absolute reduction in microglial number, that was significantly lower than ischemia-48 h hypothermia and not significantly different from sham controls (P = 0.05).

**Figure 6:**
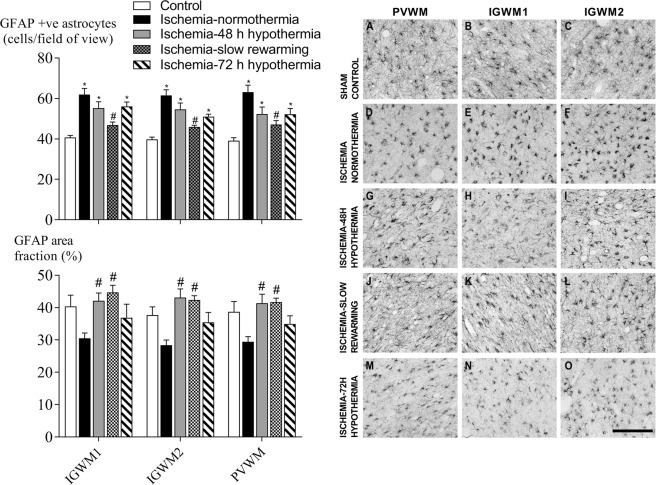
Glial-fibrillary-acidic protein (GFAP)-positive astrocyte number (top left) and area fraction (bottom left) and photomicrographs (right) in the periventricular white matter (**A**,**D**,**G**,**J**,**M**) and the intragyral white matter of the first (**B**,**E**,**H**,**K**,**N**) and second parasagittal gyri (**C**,**F**,**I**,**L**,**O**) in the sham control (**A**–**C**, n = 9), ischemia-normothermia (**D**–**F**, n = 8), ischemia-48 h hypothermia (**G**–**I**, n = 8), ischemia-slow rewarming (**J**–**L**, n = 7) and ischemia-72 h hypothermia (**M**–**O**, n = 8) groups. Data are mean ± SEM. *P < 0.05 vs sham control. #P < 0.05 vs ischemia-normothermia. Scale bar 200 µm.

Ischemia was associated with a significant overall increase in the number of GFAP positive astrocytes (P < 0.05) but a trend towards reduced total area fraction (P = 0.068) in all white matter regions compared to sham control (Fig. 6). Ischemia-slow rewarming was associated with a significant overall reduction in numbers of GFAP-positive astrocytes in all white matter regions compared to ischemia-normothermia, while the ischemia-48 h and ischemia-72 h hypothermia groups remained significantly higher than sham control (P < 0.05). The GFAP area fraction was significantly increased in the ischemia-48 h hypothermia and ischemia-slow rewarming groups compared to the ischemia-normothermia group, while an intermediate effect was seen in the ischemia-72 h hypothermia group, which was not significantly different to sham control or ischemia-normothermia.

## Discussion

The present study shows that duration and rate of rewarming have differential effects on the recovery of white matter after cerebral ischemia in near-term fetal sheep. Very slow rewarming after 48 hours of hypothermia was associated with modest effects on histological white matter recovery compared with both 48 and 72 hours of hypothermia with rapid rewarming, including better normalization of numbers of astrocytes and area fraction. However, the total duration of hypothermia and rate of rewarming after normothermia had no effect on total numbers of oligodendrocytes or the expression of myelin basic protein. Nevertheless, compared to hypothermia for 48 hours, hypothermia continued for 72 hours with rapid rewarming was associated with better recovery of mature oligodendrocytes and a greater reduction in Iba-1 positive microglia, but slightly reduced recovery of the area fraction of GFAP-positive astrocytes.

White and grey matter injury on magnetic resonance imaging (MRI) are very common after HIE and both are highly associated with subsequent neurodevelopmental impairment^15^. There is evidence from a cohort study that therapeutic hypothermia can improve fractional anisotropy on MRI compared to non-cooled infants, suggesting that hypothermia can reduce injury to the anterior and posterior limbs of the internal capsule, the corpus callosum, and the optic radiation^16^. In many infants, there was persisting white matter injury despite receiving therapeutic hypothermia, consistent with the partial white matter protection seen in all hypothermia groups in the present study. Moreover, this residual injury was associated with adverse neurodevelopmental outcomes^17^, suggesting that developing or refining interventions to further alleviate white matter injury is very important.

The optimal speed of rewarming for infants with HIE after therapeutic hypothermia to reduce white matter injury is unknown. The randomized clinical trials of therapeutic cooling for HIE typically rewarmed neonates at no more than 0.5 °C per hour^18,19^. However, this was based on limited evidence from case studies suggesting that fast rewarming might lead to hypotension or rebound seizures^20,21^, and empirical concerns arising from preclinical studies that it might reverse the depression of potentially injurious processes^22,23^. There is only limited evidence from animal studies that speed of rewarming after prolonged hypothermia can materially affect neurological outcomes. In neonatal piglets exposed to hypoxia-ischemia, 18 hours of hypothermia followed by rewarming at 0.5 °C/h was associated with less caspase-3 activation in the cerebral cortex and white matter tracts compared to rewarming at 4 °C/h^24,25^. However, 18 hours is a very short, highly suboptimal duration of hypothermia. There is now considerable preclinical evidence from both adult and neonatal studies that following delayed initiation of treatment, hypothermia must be continued for approximately 72 hours to achieve maximal neuroprotection^4,26^. Thus, as these previous studies did not control for total duration of cooling^24,25^, it is likely that the apparent reduction in white matter injury after slow rewarming was mediated by the increased total duration of therapeutic hypothermia.

We have recently shown that 48 hours of hypothermia after ischemia in near-term fetal sheep was associated with partial neuroprotection in the cortex and hippocampus that was not improved with slow rewarming, whereas neuronal survival was restored to near sham control levels when the duration of hypothermia was increased to 72 hours^11^. In the present study, by contrast, the partial protection of total numbers of oligodendrocytes seen after 48 hours of hypothermia, was not affected by either the rate of rewarming or by a longer duration of hypothermia. There was a small improvement in the survival of mature oligodendrocytes with 72 hours of hypothermia but not with slow rewarming after 48 hours of hypothermia. Given that there was no improvement in the total number of oligodendrocytes, this suggests that the longer duration of hypothermia may have been associated with improved oligodendrocyte maturation rather than greater survival.

Interestingly, oligodendrocyte survival appears to be more sensitive than neuronal survival to greater delay in starting hypothermia rather than to the duration or rate of rewarming after hypothermia. For example, we have previously shown that hypothermia started at 90 minutes after ischemia was highly protective for oligodendrocytes and neurons but when the start of hypothermia was delayed until 5.5 hours there was loss of oligodendrocyte protection despite significant, albeit partial, neuronal protection^10,13,27^. Extending the duration of hypothermia from 48 to 72 or 120 hours did not affect total numbers of oligodendrocytes but significantly modulated neuronal survival^4,9,28^.

In the present study, there was no effect of the rate of rewarming on the expression of the myelin proteins MBP or CNPase. Interestingly, 72 hours of hypothermia was associated with a small decrease in the area fraction of CNPase expression compared to the ischemia-48 hour hypothermia and ischemia-slow rewarming groups. It is not clear why CNPase expression was decreased in the ischemia-72 hour hypothermia group given the increase in mature myelinating oligodendrocytes in this group. Further, the implications of this small reduction in CNPase on the structure and function of the myelin are not known.

Consistent with many previous studies, cerebral ischemia was associated with a marked increase in the number of Iba-1 positive microglia in all white matter regions, e.g.^4,9^. All hypothermia protocols significantly reduced numbers of microglia. However, 72 hours of hypothermia with rapid rewarming was associated with a greater reduction in microglial number compared to 48 hours of hypothermia. The slow rewarming group showed an intermediate number of microglia that was not significantly different to 72 or 48 hours of hypothermia. This suggests that any benefit seen in the slow rewarming group is likely attributable to the greater duration of hypothermia rather than a true effect of the rate of rewarming per se.

Ischemia was associated with a significant increase in the number of GFAP positive astrocytes but an apparent trend to reduced area fraction of labelling (P = 0.068), consistent with a change in the morphology of astrocytes. Only ischemia-slow rewarming was associated with a significant reduction in numbers of astrocytes compared to ischemia-normothermia, while both the ischemia-48 h and ischemia-72 h hypothermia protocols had no effect on astrocyte number. Both the ischemia-48 h hypothermia and ischemia-slow rewarming groups showed a significantly greater area fraction of GFAP labelling compared to ischemia-normothermia, whereas the ischemia-72 hour hypothermia group showed an intermediate effect that was not significantly different to ischemia-normothermia or sham control. Structural remodeling of astrocytes has previously been shown 72 hours after hypoxia-ischemia in the neonatal pig brain, whereby injured astrocytes showed loss of the complex arbor of fine processes that normally extend out from the cell body of a healthy astrocyte, leading to reduced area fraction of labelling^29^. These data suggest that in the current study ischemia was associated with an increase in the number of astrocytes but loss of these fine processes. The slow rewarming protocol best restored both astrocyte number and area fraction of GFAP labelling, suggesting improved astrocyte morphology. Given that the slow rewarming protocol was more effective than either 48 hours or 72 hours of hypothermia with rapid rewarming, this suggests a true benefit of slow rewarming on astrocytosis, rather than an effect of prolonging the duration of hypothermia.

We have previously shown that the slow rewarming protocol was associated with a significant improvement in the recovery of EEG power compared to 48 hours of hypothermia with rapid rewarming, despite slow rewarming having no effect on neuronal survival^11^. Thus, we speculate that secondary astrocytosis may contribute to electrophysiological recovery. Astrocytes play a critical role in maintaining homeostasis in the brain, including uptake and recycling of extracellular glutamate, buffering of potassium ions and modulation of synaptic transmission via their contribution to the “tripartite synapse” as reviewed^30^. Further, in the present study, we only assessed changes in white matter astrocytes. However, similar astrocytic structural remodeling after hypoxia-ischemia has been shown in grey matter astrocytes in the neonatal pig brain^31^. It is plausible that if the slow rewarming protocol showed superior protection of grey matter astrocytes, this may also contribute to the improved recovery of EEG power seen after slow rewarming^11^.

## Conclusion

The effect of rate of rewarming after hypothermia and duration of hypothermia in white matter recovery from global cerebral ischemia are complex and cell type specific. Very slow rewarming at a rate of 0.2 °C per hour after 48 hours of hypothermia was associated with modestly improved normalization of astrocyte number and area fraction compared to either 48 or 72 hours of hypothermia with rapid rewarming. However, there was intermediate improvement in Iba-1 positive microglia with slow rewarming after 48 hours of hypothermia compared with rapid rewarming after 48 hours and 72 hours of hypothermia, consistent with an effect of prolongation of the duration of hypothermia rather than the rate of rewarming *per se*. Moreover, the duration of hypothermia and rate of rewarming after normothermia had no effect on total numbers of oligodendrocytes or preservation of MBP. Finally, hypothermia for 72 hours with rapid rewarming was associated with the greatest recovery of mature oligodendrocytes albeit with a small reduction in recovery of the area fraction of CNPase labelling. Overall, pragmatically, these data suggest that the net duration of hypothermia has a more important impact on hypoxic-ischemic white matter injury than speed of rewarming after therapeutic hypothermia.
